# A single dose of eHSP72 attenuates sepsis severity in mice

**DOI:** 10.1038/s41598-020-66011-y

**Published:** 2020-06-08

**Authors:** Maicon Machado Sulzbacher, Lucas Machado Sulzbacher, Felipe Rafael Passos, Bruna Letícia Endl Bilibio, Wellington Felipe Althaus, Luana Weizenmann, Kauana de Oliveira, Matias Nunes Frizzo, Mirna Stela Ludwig, Thiago Gomes Heck

**Affiliations:** 1 Research Group in Physiology, Department of Life Sciences, Regional University of Northwestern Rio Grande do Sul State (UNIJUÍ), Ijuí, RS Brazil; 2 Postgraduate Program in Integral Attention to Health (PPGAIS-UNIJUÍ/UNICRUZ), Ijuí, RS Brazil

**Keywords:** Neurological disorders, Physiology, Chaperones

## Abstract

High levels of extracellular 72 kDa heat shock protein (eHSP72) can be detected in the serum of septic patients and are associated with increased oxidative profiles and elevated rates of mortality among these patients. However, a possible immunomodulatory role for this protein, resulting in tissue protection during sepsis, has never been assessed. In this study, we investigated whether eHSP72 administration could attenuate the severity of sepsis in a mouse peritonitis model. Animals (90-day-old male C57BL/6J mice) were divided into Sepsis (n = 8) and Sepsis + eHSP72 (n = 9) groups, which both received injections of 20% fecal solution [1 mg/g body weight (wt), intraperitoneal (i.p.)], to trigger peritonitis induced-sepsis, whereas a Control group (n = 7) received a saline injection. eHSP72 was administered (1.33 ng/g body wt) to the Sepsis+eHSP72 group, 12 h after sepsis induction. All animals were evaluated for murine sepsis score (MSS), hemogram, core temperature, and glycemia (before and 4, 12, and 24 h after sepsis induction). Treatment with eHSP72 promoted reduced sepsis severity 24 h after sepsis induction, based on MSS scores (Control = 1.14 ± 1.02; Sepsis = 11.07 ± 7.24, and Sepsis + eHSP72 = 5.62 ± 1.72, *P* < 0.001) and core temperatures (°C; Control = 37.48 ± 0.58; Sepsis = 35.17 ± 2.88, and Sepsis + eHSP72 = 36.94 ± 2.02; *P* = 0.006). eHSP72 treatment also limited the oxidative profile and respiratory dysfunction in mice with sepsis. Although sepsis modified glycemic levels and white and red blood cell counts, these variables were not influenced by eHSP72 treatment (*P* > 0.05). Finally, eHSP72 improved the survival rate after sepsis (*P* = 0.0371). Together, our results indicated that eHSP72 may ameliorate sepsis severity and possibly improve some sepsis indices in mice.

## Introduction

Approximately 30 million patients are affected by sepsis each year, resulting in approximately 6 million deaths^1^. Sepsis is defined as life-threatening organ dysfunction, caused by a dysregulated host response to infection, which damages the normal functions of the immune, respiratory, cardiovascular, renal, and central nervous systems, and impacts coagulation, body temperature control, and metabolic homeostasis^2^. Altered levels of the 72 kDa heat shock protein (HSP72), observed in extracellular fluids (eHSP72), such as plasma, serum, and liquor, and intracellular (iHSP72) milieu, such as leukocytes^3^, are frequently accompanied by an oxidative stress profile^4^ and have been associated with the severity and mortality of sepsis in both human^2,4,5^ and animal models^6,7^.

High eHSP72 serum concentrations have been detected in septic patients^4,8–11^. Additionally, eHSP72 levels have been observed at increased levels in septic patients compared with systematic inflammatory response syndrome (SIRS) patients^9^. Furthermore, elevated eHSP72 levels may represent a strong predictor of mortality^8,10^.

eHSP72 has been proposed to play relevant roles against infections, potentially through immunomodulatory functions^12–15^. During homeostatic challenges, eHSP72 signals physiological systems^13,16^ by binding to Toll-like receptors (TLR), such as TLR-4, in a variety of cells, which activates pro-inflammatory pathways. The resulting activation of myeloid differentiation primary response protein (MyD88) and Toll/interleukin-1 receptor domain-containing adaptor protein (TIRAP) promotes nuclear factor κB (NF-κB) signaling and the activation of c-Jun amino-terminal kinases (JNKs)^13^. Thus, eHSP72 stimulates immunoinflammatory systems^12^, increasing microbicide capacity^17^ and the chemotaxis of neutrophils^18^, while simultaneously boosting cytokine release from various immune cells^14,19^, which eventually trigger the pro-inflammatory responses that elevate immunosurveillance^15^. Interestingly, when elevated eHSP72 levels are related to increased mortality^4^ when associated with an increased oxidative profile in the serum of sepsis patients, which may result from the loss of immune signaling properties, due to eHSP72 oxidation^20^.

In white blood cells obtained from patients, Briassoulis *et al*.^21^ demonstrated that iHSP72 protein synthesis was activated during sepsis, as iHSP72 protein expression was increased in septic patients compared with that in healthy individuals. However, when septic patients were compared with systemic inflammatory response syndrome (SIRS) patients, lower iHSP72 levels were observed in monocytes^10,11^ and neutrophils from septic patients than in SIRS patients^10^. Similarly, neutrophil iHSP72 levels were lower in septic shock patients than in non-septic shock patients^8^.

Because enhanced iHSP72 levels may be associated with improved host defense, the failure of this cytoprotective heat shock response in immune cells may be associated with cytokine burst signaling observed during sepsis^22,23^. Reduced iHSP72 expression in monocytes in response to a lipopolysaccharide (LPS) insult was associated with a cytokine burst^21^. Furthermore, repressed iHSP72 expression has been associated with an increased mortality rate and the repressed expression of CD14/HLA-DR in monocyte and polymorphonuclear cells during sepsis^23^. Additionally, iHSP72 may counteract nuclear factor (NF)-κB activation and the associated burst of pro-inflammatory cytokine signalling^24^. Because eHSP72 may be internalized by antigen-presenting (and other) cells^25^, eHSP72 may be able to act as an authentic iHSP72 and induce a similar protective response.

Therefore, eHSP72 may represent a beneficial factor that can re-establish immunoinflammatory balance during sepsis, which has never before been assessed, although the administration of recombinant eHSP72 has been found to increase survival rates in septic rats^6,26^. Therefore, we aimed to investigate whether the intravenous administration of eHSP72 might attenuate the severity of sepsis in a mouse peritonitis model.

## Materials and Methods

### Animals and ethics

Male, C57BL/6J mice (n = 24), aged 3–5-months, were obtained from the Regional University of the Northwestern Rio Grande do Sul State (UNIJUÍ) Animal Facility and maintained in a controlled-temperature environment (24 ± 2 °C), with a 12-h light-dark cycle (light from 7:00 a.m. to 7:00 p.m.). The animals received water and food *ad libitum*. The investigation followed all ethical rules established by Arouca’s Act (Brazilian Federal Law no. 11794/2008) and the 8th Edition of the Guide for Care and Use of Experimental Animals, published by the National Research Council of the National Academies (2011; available at https://grants.nih.gov/grants/olaw/guide-for-the-care-and-use-of-laboratory-animals.pdf), in accordance with Animal Research: Reporting of *In Vivo* Experiments (ARRIVE) guidelines. All the procedures were reviewed and approved by the Committee of Animal Welfare of the Regional University of the Northwestern Rio Grande do Sul State (UNIJUÍ; protocol CEUA-UNIJUI #048/2016), which adheres to the guidelines of The Brazilian National Council for the Control of Animal Experimentation (CONCEA).

### Experimental design

Mice (n = 24) were randomly divided into three groups: Control (n = 7), Sepsis (n = 8) and Sepsis + eHSP72 (n = 9). Animals from Sepsis and Sepsis + eHSP72 groups received an intraperitoneal (i.p.) injection of 20% fecal solution [1 mg/g body weight (wt), containing a bacterial load of approximately 43 and 99 CFUs/g of *Escherichia coli* and *Staphylococcus aureus*, respectively, as detailed in Supplementary Material 1] to trigger peritonitis induced-sepsis, whereas the Control group received 0.9% saline (i.p.)^27^. Then, 12 h after sepsis induction, eHSP72 (hspa1a, mouse recombinant low-endotoxin, Enzo ADI-ESP-502, 98.4% homologous with *Rattus norvegicus* hspa1a) was administered intravenously (i.v.) (1.33 ng/g) to mice in the Sepsis + eHSP72 group. We choose this dose because mice with 30 g of body weight contain approximately 2 ml of blood^28,29^; thus the concentration of eHSP72 in the blood was expected to reach approximately 20 ng/ml, which was the concentration reported for an experimental mouse model of sepsis 24 h after cecal ligation puncture (CLP)^30^. We administered eHSP72 12 h after sepsis induction, in an attempt to anticipate the immune-mediated responses of eHSP72. All animals were evaluated by recording the murine sepsis score (MSS), glycemia, hemogram, and rectal temperature at the following times: just before group assignment (time 0 h), 4, and 12 h after fecal solution administration for sepsis induction (before eHSP72 administration), and 24 h after sepsis induction. No animal deaths were recorded up to this time point, but the mice were followed up to 48 h after sepsis induction, for the assessment of survival rates (Kaplan-Meier).

### Murine sepsis score test

The murine sepsis score (MSS) included variables affected by the infection, including spontaneous activity, response to touch and auditory stimuli, posture, respiration rate and quality (labored breathing or gasping), and appearance (the degree of piloerection). For each of these variables, a score between 0 and 4 was given (See details in ref. ^31^). Two independent, blinded researchers, who were previously trained, analyzed the videos.

### Bodyweight, glycemia, and core temperature

Individual animal body weights were assessed using a semi-analytical scale, just before group assignments (time 0 h), 4, and 12 h after fecal solution administration for sepsis induction. At the same time points, blood glucose levels were measured, using a Glucometer Optium Xceed (Abbott) (5 µL of tail blood)^32^, and the core temperatures of the animals were assessed using a Minipa Digital Thermometer MT450, equipped with a rectal probe^24^.

### Hematological parameters

Blood samples were collected from each animal by caudal puncture (10 µl), just before group assignment (time 0 h), 4, and 12 h after the administration of fecal solution for sepsis induction. were diluted 1:3 with 0.9% saline, containing 1 µl of anticoagulant [Ethylenediaminetetraacetic acid (EDTA)]. Afterward, slides were subjected to hematological staining to obtain leukocyte counts (neutrophils, lymphocytes, and monocytes)^32^, by using panoptic type staining (Newprov). Smears were analyzed by a professional with experience in the field to confirm automated data acquisition^33^. For automatic determination, a Micros 60 hematology analyzer (Horiba) was used, following the manufacturer’s recommendations. The following parameters were obtained: hematocrit level, total leukocyte count, absolute leukocyte count, plus platelet count, platelet count, and mean platelet volume (Horiba-User Manual).

### Oxidative stress and antioxidant enzyme activity

Lungs were collected at the end of experiments (48 h) and lipid peroxidation was evaluated using the thiobarbituric acid reactive substances (TBARS) method^34^. Homogenates were precipitated with 10% trichloroacetic acid, for 30 min on ice, centrifuged (3000 rpm, 10 min), and incubated with thiobarbituric acid (TBA), for 15 min at 100 °C. Then, the absorbance was measured at 535 nm. The protected form of malondialdehyde (MDA), 1,1,3,3-Tetramethoxypropane, was used as the standard, at concentrations between 0.0005 and 0.016 mg/mL. The results are expressed as mmol MDA equivalents/g of tissue. Lung superoxide dismutase (SOD) activity was analyzed by inhibiting the auto-oxidation of pyrogallol^35^. Briefly, in a cuvette, 954 μL 50 mM Tris/1 mM EDTA buffer (pH 8.2), 4 μL catalase (30 µM), 10 µL lung homogenate, and 32 µL pyrogallol (24 mM in 10 mM HCl) were combined and mixed. SOD activity was determined at 36 °C, in a spectrophotometer (420 nm), every 20 s for 120 s. The results are expressed as the inhibition percentage of pyrogallol auto-oxidation.

### Intracellular HSP70 (iHSP70) tissue expression

iHSP70 expression was evaluated in the lungs by immunoblot analyses, as described previously^16^. Briefly, equivalent amounts of protein from each sample (approximately 40 µg) were mixed with Laemmli’s gel loading buffer [50 mM Tris, 10% (w/v) SDS, 10% (v/v) glycerol, 10% (v/v) 2-mercaptoethanol, and 2 mg/mL bromphenol blue], at a ratio of 1:1, boiled for 10 min, and electrophoresed in a 10% polyacrylamide gel (5 h at 15 mA/gel). The proteins were then transferred onto a nitrocellulose membrane (GE Healthcare) by electrotransfer (1 h in 100 V), and, subsequently, the transferred bands were visualized with 3% (w/v) Red Ponceau S (Sigma-Aldrich). Membranes were washed with a TEN (50 mM Tris, 5 mM EDTA, 150 mM NaCl, pH 7.4)-Tween 20 solution [0.1% (w/v)] and then blocked in 5% (w/v) non-fat dry milk in washing buffer (TEN-Tween 20 solution). Membranes were incubated for 12 h with the monoclonal anti-hsp70 antibody (Sigma-Aldrich H5147, 1:1,000) which recognizes both the constitutive (hspa8) and inducible (hspa1a) forms of hsp70. After three consecutive washes with the washing buffer, peroxidase-labeled rabbit anti-mouse IgG (Sigma-Aldrich A9044) was utilized as a secondary antibody, at 1:50,000 dilution. As a gel loading control, we used the detection of the 43 kDa β-actin (A3854 antibody Sigma-Aldrich, 1:50,000). Blot visualization was performed using ECL-Prime Western Blotting Reagent (GE Healthcare). The quantification of band intensity was performed using Image J software. The data are presented in arbitrary units of iHSP70/β-actin.

### Statistical analysis

All data were verified for normality by the Kolmogorov-Smirnov test, and results are expressed as the mean ± standard deviation. Comparisons among groups and time effects were analyzed by two-way analysis of variance (ANOVA, treatment × time), followed by the Bonferroni *post hoc* test. Data obtained at the end of the study, such as respiratory frequency, oxidative stress parameters, and HSP70 expression, were analyzed by one-way ANOVA, followed by the Tukey *post hoc* test. All statistical analyses were performed using GraphPad for Windows, version 8.0. The level of significance was set to *P* < 0.05. Our experimental approach had a 70% power to detect a survival difference of 0.630, with a significance level (alpha) of 0.05 (two-tailed), as calculated in StatMate Software 2.0 (GraphPad). Kaplan-Meier estimates of cumulative survival were generated (GraphPad) and assessed for standard errors and 95% confidence intervals, using the Cox-Mantel rank test and a Chi-squared value, from which the *P*-values were obtained.

## Results

**Figure 1:**
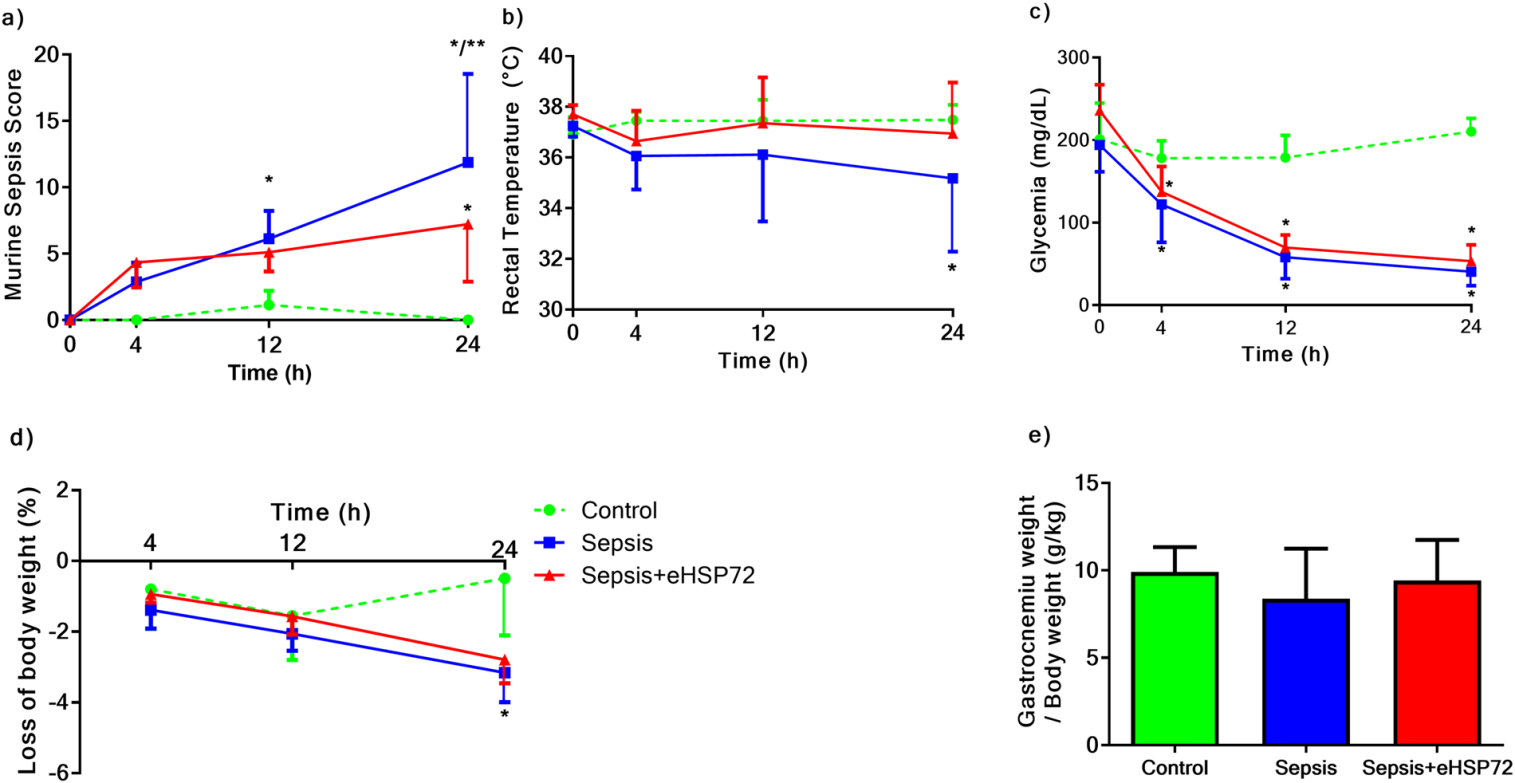
Effects of eHSP72 administration on murine sepsis score (MSS), rectal temperature, glycemia, and biometric profile, in mice with sepsis. Sepsis was induced by the administration of a 20% fecal solution (1 mg/g, i.p.), whereas the Control group (n = 7) received saline. eHSP72 was administered (1.33 ng/g) to the Sepsis + eHSP72 group,12 h after sepsis induction (n = 9), whereas the Sepsis group received saline (n = 8). MSS (**a**), rectal temperature (**b**), glycemia (**c**), loss of body weight (**d**), and muscle mass (**e**). A two-way ANOVA, followed by Bonferroni *post hoc* test, was used in A, B, C, and D. One-way ANOVA, followed by the Tukey test, was used in E. ^*^P < 0.05 vs. Control; ^**^P < 0.05 Sepsis vs. Sepsis + eHSP72.

Animals submitted to sepsis showed increased MSS values both 12 h and 24 h after sepsis induction. However, the severity of sepsis evaluated by MSS was lower 24 h after sepsis induction in mice treated with eHSP72 (Fig. 1a). Sepsis also induced a decrease in mouse body temperatures compared with the Control group; however, eHSP72 administration reduced this effect (Fig. 1b). Furthermore, sepsis-induced a decrease in glycemic levels and body weights compared with the Control group, and eHSP72 treatment did not influence these effects (Fig. 1c,d). We did not observe any relative muscle mass alterations, as assessed by comparisons of the gastrocnemius muscle to body weight ratios, among the groups (Fig. 1e).

**Figure 2:**
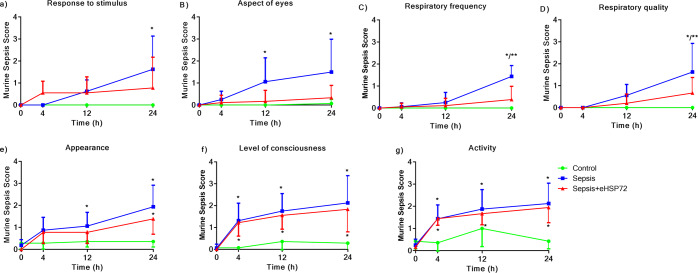
Effects of eHSP72 administration on individual components of the murine sepsis score (MSS). Experimental conditions were those described for Fig. 1 Response to a stimulus (**a**), aspect of eyes (**b**), respiratory frequency (**c**), respiratory quality (**d**), appearance (**e**), level of consciousness (**f**), and activity (**g**). A two-way ANOVA, followed by Bonferroni *post hoc* test, was used. ^*^P < 0.05 vs. Control; ^**^P < 0.05 Sepsis vs. Sepsis + eHSP72.

The behaviors of each variable evaluated to determine the total MSS, which is shown in Fig. 1, can be observed in detail in Fig. 2. Sepsis worsened all measured variables compared with those in the Control group, including the response to stimulus, eye-related aspects, the respiratory rate, the quality of respiration, appearance, levels of consciousness, and motor activity (Fig. 2a–g). The eHSP72-treated group presented better MSS scores than the Sepsis group in response to the stimulus test, eye-related aspects, the respiratory rate, and the quality of respiration (Fig. 2a–d), whereas levels of consciousness and motor activity were not modified (Fig. 2e–g).

**Figure 3:**
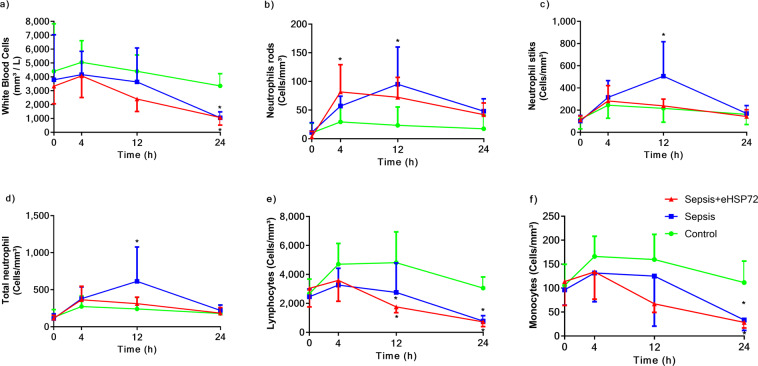
Effects of eHSP72 administration on leukocyte counts in mice with sepsis. Experimental conditions were those described for Fig. 1. White blood cells (**a**), neutrophils rods (**b**), neutrophil sticks (**c**), total neutrophils (**d**), lymphocytes (**e**), and monocytes (**f**). A two-way ANOVA, followed by Bonferroni post hoc test was used. ^*^P < 0.05 vs. Control; ^**^P < 0.05 Sepsis vs. Sepsis + eHSP72.

**Figure 4:**
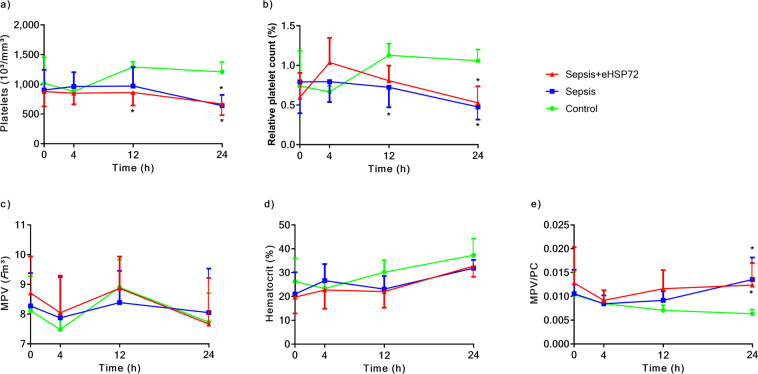
Effects of eHSP72 administration on platelets and hematocrit in mice with sepsis. Experimental conditions were those described for Fig. 1. Platelets (**a**), relative platelets (**b**), MPV (**c**), hematocrit (**d**), and MPV/PC (**e**). A two-way ANOVA, followed by Bonferroni *post hoc* test was used. ^*^P < 0.05 vs. Control; ^**^P < 0.05 Sepsis vs. Sepsis + eHSP72.

We evaluated the hematological profiles of mice for 24 h. Total leukocyte counts decreased in mice with sepsis, 24 h after fecal administration, which were not affected by eHSP72 treatment (Fig. 3a). Additionally, animals with sepsis presented increased neutrophil counts (Fig. 3b–d) and decreased lymphocyte (Fig. 3e) and monocyte (Fig. 3f) counts. Sepsis also induced decreases in both the absolute (Fig. 4a) and relative (Fig. 4b) platelet counts, without affecting mean platelet volume (Fig. 4c) or hematocrit levels (Fig. 4d). Thus, sepsis promoted and increased the mean platelet volume to platelet count ratio (MPV/PC) (Fig. 4e).

**Figure 5:**
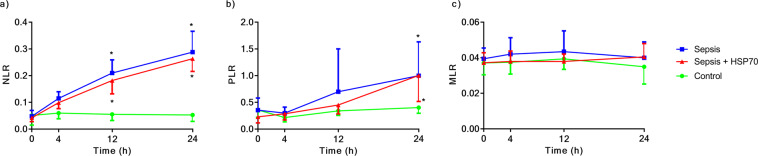
Effects of eHSP72 administration on the ratio of blood cells in mice with sepsis. Experimental conditions were those described for Fig. 1. Neutrophil-to-lymphocyte ratio (**a**), platelets-to-lymphocyte ratio (**b**) and monocyte-to-lymphocyte ratio (**C**). Two-way ANOVA, followed by Bonferroni post hoc test was used. ^*^P < 0.05 vs. Control; ^**^P < 0.05 Sepsis vs. Sepsis + eHSP72.

To gain further insight into the inflammatory response during sepsis, we analyzed the equilibrium among blood cells counts. At 12 and 24 h after sepsis induction, an inflammatory response was observed, as indicated by an increase in the neutrophil-to-lymphocyte ratio (NLR) (Fig. 5a), which was associated with an increase in the platelet-to-lymphocyte ratio (PLR) at 24 h (Fig. 5b). We did not observe any alterations in the monocyte-to-lymphocyte ratio (MLR) (Fig. 5c). Treatment with eHSP72 did not influence these responses.

**Figure 6:**
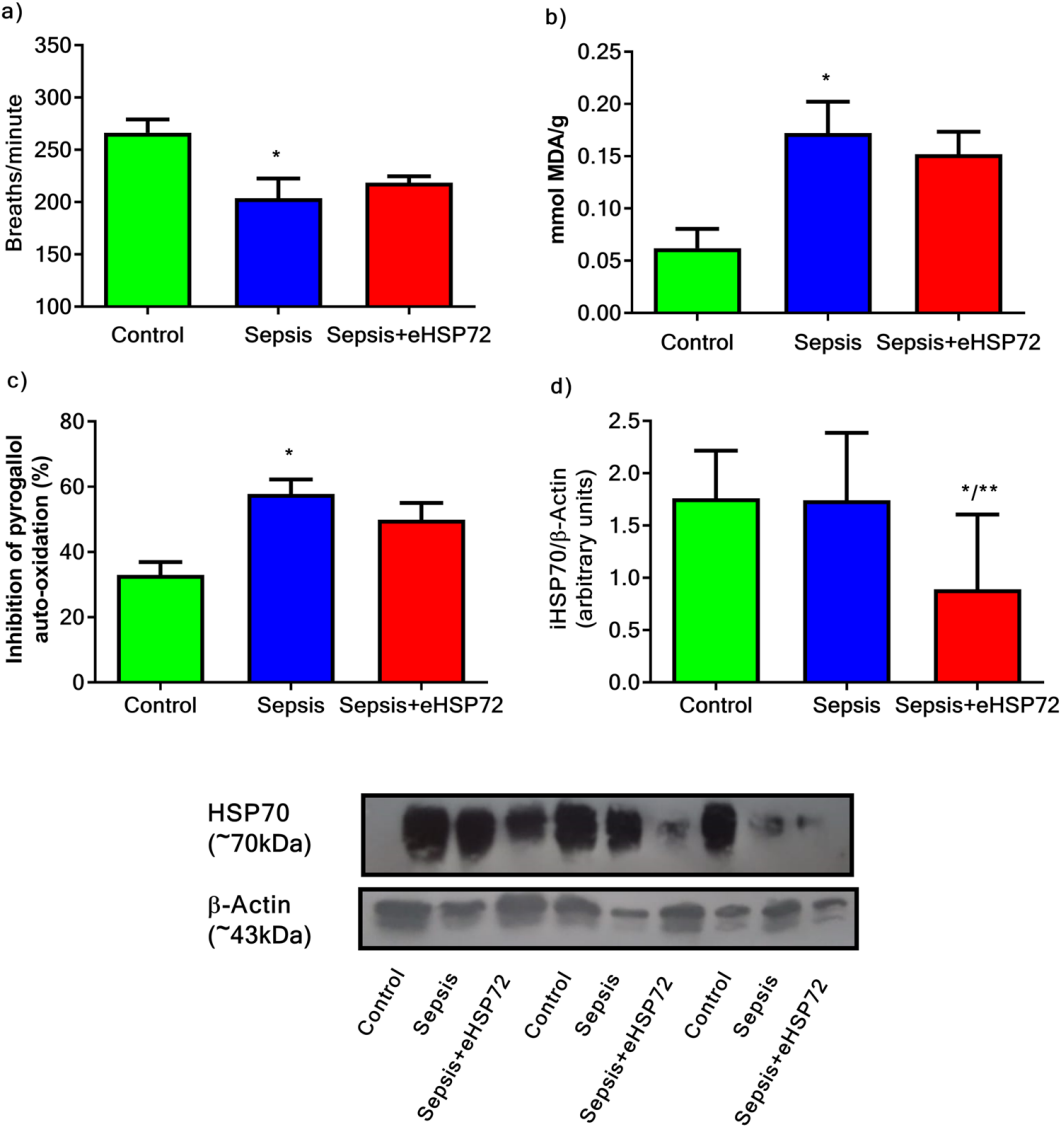
Effects of eHSP72 administration on lung function, oxidative, and cell stress biomarkers in mice with sepsis. Experimental conditions were those described for Fig. 1. Respiratory frequency (**a**), lung lipid peroxidation levels (**b**), SOD activity (**c**), and 70 kDa heat shock protein (HSP70) expression (**d**). Two-way ANOVA, followed by Bonferroni post hoc test was used. ^*^P < 0.05 vs. Control; ^**^P < 0.05 Sepsis vs. Sepsis+eHSP72. Representative blots are shown at the bottom.

We observed decreased respiratory rates in mice, 24 h after sepsis induction (Fig. 6a), accompanied by increased oxidative profiles in the lungs, as demonstrated by increased lung lipid peroxidation levels (Fig. 6b) and SOD activity levels (Fig. 6c) in the Sepsis group compared with those in the Control group. Interestingly, the Sepsis + eHSP72 group showed attenuated effects among these parameters and decreased HSP70 levels were observed in the eHSP72-treated group relative to those in the Sepsis group (Fig. 6d).

**Figure 7:**
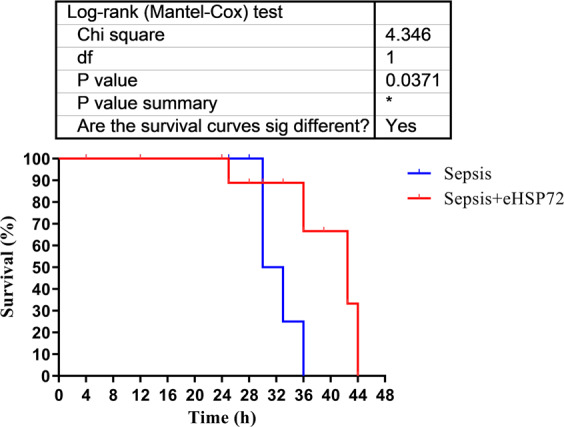
Effects of eHSP72 administration on the survival rate of mice with sepsis. Experimental conditions were those described for Fig. 1. Kaplan-Meier statistics showed an increase in the survival time for the eHSP72 + Sepsis group compared with the Sepsis group (*P* = 0.0371, Chi-squared test).

Finally, we performed a separate experiment to evaluate the effects of eHSP72 administration on cumulative survival. An increase in the survival rate was observed for the eHSP72 + Sepsis group compared with that for the Sepsis group (*P* = 0.0371) (Fig. 7). All animals from the Sepsis group died within the first 36 h after sepsis induction, whereas, during the same time period, the survival rate in the Sepsis+eHSP72 group was 90%. However, 70% of the mice in the Sepsis+eHSP72 group died within 42 h after sepsis induction (Fig. 7).

## Discussion

In our study, we demonstrated that eHSP72 treatment, when used during the early stages of sepsis, attenuated sepsis severity in an animal model, both clinical and subclinically. eHSP72 administration promoted positive effects on the survival rate, responsiveness, and respiratory functions, which were related to the attenuation of the lung oxidative profile. Although some variables were not improved by eHSP72, none of the variables was worsened by this treatment in the mouse model of sepsis.

We believe that eHSP72 administration may mimic the roles played by alarmins or danger signals (danger-activated molecular patterns or DAMPs)^25,36^, due to its interactions with pattern recognition receptors, such as TLR2, TLR4, lectin-like oxidized low-density lipoprotein receptor-1 (LOX-1), CD91, CD94 (C-type lectin), CD40, and chemokine receptor CCR5, resulting in DAMP-like signaling^25,36,37^. eHSP72 can bind to a wide repertoire of cell-surface receptors that may result in internalization^38,39^; therefore, eHSP72 administration may induce intracellular responses. Additionally, cell surface molecules of the interrelated scavenger receptor and C-type lectin families may also internalize eHSP72^39^. At least three members of the scavenger receptor family are able to bind HSP72-peptide complexes and mediate efficient internalization: LOX-1, fasciclin, epidermal growth factor-like, laminin-type epidermal growth factor-like, and link domain-containing scavenger receptor-1 (Stabilin-1); and scavenger receptors expressed by endothelial cells-1 (SREC-1). In addition, eHSP72 internalization may occur independently of the intracellular domains of scavenger receptors, as eHSP72 protein transport can occur even after the deletion LOX-1 or SREC-1 intracellular domain, as observed by the internalizing receptor CD91/LRP1 HSP-mediated cross-presentation. Furthermore, evidence suggests that a cooperative system exists to internalize HSPs, as LOX-1-mediated Hsp70 internalization is markedly reduced in CD91/LPR1-deficient cells^39^.

By binding cell-surface receptors, eHSP72 administration can modify intracellular cell signaling, providing anti-inflammatory benefits. eHSP72 has been demonstrated to reverse inflammatory imbalances through mitogen-activated protein kinase (MAPK) pathways, as described in rodent brain structures^40^. The intrahippocampal infusion of eHSP72 was also shown to enhance MAPK pathways by increasing the activity of extracellular signal-regulated kinase (ERK) and decreasing the activity of protein kinase 38 (p38) and JNK^40^. eHSP72 administration also promotes cardiovascular benefits during sepsis^6^, which may act to preserve cerebral hemodynamics and account for the observed improvements in the MSS evaluations that were observed in our study in eHSP72+Sepsis mice. Sepsis often results in central nervous system dysfunction; therefore, our MSS results, particularly those for the consciousness levels of mice, may indicate the potential neuroprotective function of eHSP72 treatment against infection-related dysfunction^41^. These results agree with the findings reported Chang *et al*., who demonstrated the amelioration of sepsis-related cardiovascular dysfunction, induced by the direct action of eHSP72 in the ventrolateral rostral amygdala^42^.

The administration of eHSP72 resulted in reduced levels of the anti-inflammatory iHSP70 in the lungs of treated animals. However, the plasma levels of eHSP72 have been shown to return to basal levels only 180 min after the intravenous administration of eHSP72, which suggests the tissue uptake of eHSP72^6^. The heat shock factor-1 (HSF-1)-induced expression of iHSP72 can be blocked by excess iHSP70; therefore, the uptake and internalization of eHSP72 by immune cells^43^ may similarly decrease the expression of lung iHSP70 observed in the present study.

eHSP72 treatment also attenuated the oxidative profile induced by sepsis, in our study. Our investigation showed that eHSP72 attenuated sepsis-induced alterations in the respiratory pattern and the lung oxidative profile, which are commonly observed during peritonitis^31,44,45^. Because the oxidative burst in the lungs is related to leukocyte infiltration, especially neutrophil activity against microorganism invasion^46^, the ability of eHSP72 to attenuate respiratory dysfunction by decreasing the neutrophil- and monocyte-dependent production of reactive oxygen species (ROS) after exposure to bacterial antigens^47^ may explain the respiratory system benefits observed in our study. The reduction of vascular permeability induced by eHSP72^48,49^ may also be a plausible explanation for these findings.

In our study, eHSP72 administration attenuated the sepsis-induced decrease in body temperature by 50% (0.5 °C). Because eHSP72 may decrease NO production by leukocytes during infection, which has been associated with improved cardiovascular function in an animal sepsis model^50^, and NO is also involved in the central control of thermoregulation^51^, eHSP72 may affect mouse body temperature through the modulation of NO metabolism in the central nervous system, as previously suggested^16,51^.

In addition to animal model studies, the role of HSP72 (both eHSP72 and iHSP72) has been investigated in humans. Patients with sepsis and severe sepsis demonstrated increased serum eHSP72 compared with controls and with SIRS patients, which enables the discrimination of sepsis from SIRS in patients across all age groups^9–11^. eHSP72 levels were also detected at increased levels among non-survivors compared with survivors and are considered to be a strong predictor of mortality^8^. When sepsis patients were compared with SIRS patients, lower iHSP72 levels were found in monocytes^10,11^ and neutrophils from sepsis patients^10^.

The association between eHSP72 and mortality in sepsis appears primarily in conjunction with an oxidative profile^4^. The elevation of eHSP72 during sepsis may represent a tentative immune-related danger signal^25,36^ that fails under oxidative stress conditions, during which eHSP72 may lose its functionality, including the ability to trigger immune signaling pathways^20^.

Similarly, when eHSP72 levels increase, iHSP72 levels are repressed in septic patients^52^, and the elevated concentrations of these proteins in the bloodstream may be related to glucocorticoid receptor expression, induced by eHSP72 in monocytes, which facilitate cortisol binding to counterbalance the increased inflammatory response during early sepsis. As discussed by Vardas and colleagues^8^, exogenous steroid support may not be necessary due to signals mediated by the glucocorticoid receptor, associated with stress-activated bio-molecules and organ dysfunction. Together, these pieces of evidence suggest a positive aspect of our study, which was that eHSP72 administration that occurred 12 h after sepsis induction was able to attenuate the severity of related neurological functions, as observed during the MSS test.

However, human studies examining eHSP72 administration during sepsis have been inconclusive. Briassoulis and colleagues compared various sepsis outcomes, which differ greatly between human and animal studies^3^. Clinical studies have shown a low probability of protection (16.7%) and possible relationships with mortality and infections for eHSP72 administration. In contrast, almost all (94.7%) septic animals in both *in vivo* and *in vitro* studies showed biochemically, biologically, and clinically protective effects for eHSP72 during sepsis, in agreement with our study. Our study also demonstrated that a single dose of eHSP72 promoted benefits in a mouse model of sepsis. Further studies should be performed to provide a more complete description regarding the dose-response of eHSP72 on the immune system during sepsis and to investigate cytokine levels and the effects of eHSP72 treatment on immune cell activation, including splenocytes and peritoneal macrophages.

## Conclusion

Treatment with eHSP72 promoted benefits in the stimulus-response, body temperature control, respiratory pattern, survival rate, and oxidative profile of a mice sepsis model. Together, our results indicate that treatment with eHSP72 may attenuate the severity of sepsis in an animal model, opening a perspective for future studies against sepsis.

## Supplementary information

Supplementary information.

## Data Availability

The data used to support the findings of this study are available from the corresponding authors upon request.
