# Supplementary material for: A single dose of eHSP72 attenuates sepsis severity in mice
Source: Sci Rep. 2020 Jun 8;10:9198. doi: 10.1038/s41598-020-66011-y (PMC7280184; doi:10.1038/s41598-020-66011-y)

A single dose of eHSP72 administration attenuates sepsis severity in mice

Maicon Machado Sulzbacher[1,2*], Lucas Machado Sulzbacher[1], Felipe Rafael Passos[1], Bruna Letícia Endl Bilibio[1,2], Wellington Althaus[1], Luana Weizenmann[1], Kauana de Oliveira[1], Matias Nunes Frizzo[1,2], Mirna Stela Ludwig[1,2], Thiago Gomes Heck[1,2]

[1]Research Group in Physiology, Department of Life Sciences, Regional University of Northwestern Rio Grande do Sul State (UNIJUÍ), Ijuí, RS, Brazil.
[2]Postgraduate Program in Integral Attention to Health (PPGAIS-UNIJUÍ/UNICRUZ), Ijuí, RS, Brazil

Supplementary material 1.

Supplementary methods:

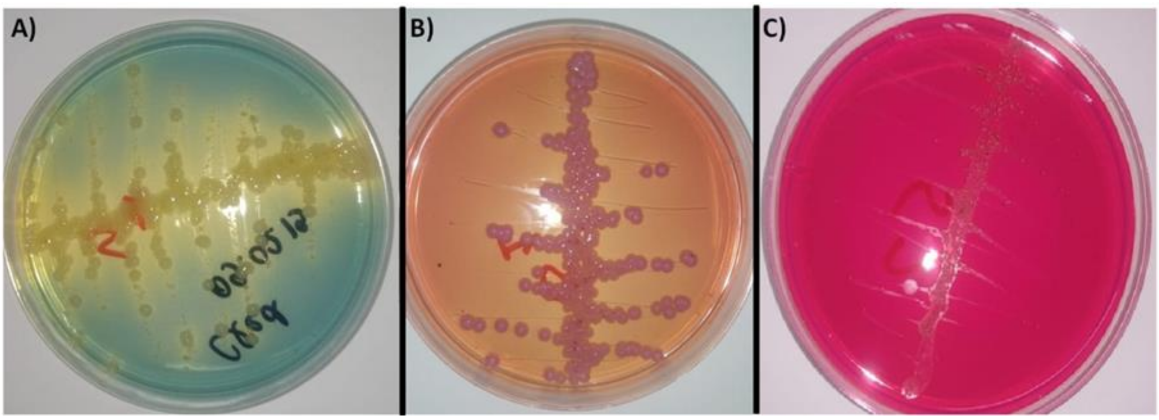

Supplementary Figure 1. Representative images from bacterial analyses of faecal solution administered in mice for sepsis induction. Faecal solution was prepared in sterile NaCl 0.9% solution (100 mg/ml). Bacterial colonies were analysed by two independent evaluators. (A) In the Agar Cled medium (INTERLAB) (A) were identified colonies of *Escherichia coli* and *Staphylococcus aureus*. Thus, the CFU count were performed in (B) Agar MacConkey (HIMEDIA) and (C) Agar Sal mannitol IO053 (IONLAB) by incubation at 35 °C (± 2) by 24 h. We found 216 ± 0.707 colonies of *E. coli* and 494 ± 9.192 of *S. aureus*. Since the sample volume used in this analysis was 50 µL, representing five milligrams of faeces and was administered at the dose of 1 mg/g per animal, we estimated that 43 CFUs/g of *E. coli* and 99 CFUs/g of *S. aureus* were i.p. administered for sepsis induction.

Murine Sepsis Score (MSS) (SHRUM et al., 2014)

Variable	Score and description
Appearance	0- Coat is smooth<br>1- Patches of hair piloerected<br>2- Majority of back is piloerected<br>3- Piloerection may or may not be present, mouse appears "puffy"<br>4- Piloerection may or may not be present, mouse appears emaciated
Level of consciousness	0- Mouse is active<br>1- Mouse is active but avoids standing upright<br>2- Mouse activity is noticeably slowed. The mouse is still ambulant.<br>3- Activity is impaired. Mouse only moves when provoked, movements have a tremor<br>4- Activity severely impaired. Mouse remains stationary when provoked, with possible tremor
Activity	0- Normal amount of activity. Mouse is any of: eating, drinking, climbing, running, fighting<br>1- Slightly suppressed activity. Mouse is moving around bottom of cage<br>2- Suppressed activity. Mouse is stationary with occasional investigative movements<br>3- No activity. Mouse is stationary<br>4- No activity. Mouse experiencing tremors, particularly in the hind legs
Response to stímulus	0- Mouse responds immediately to auditory stimulus or touch<br>1- Slow or no response to auditory stimulus; strong response to touch (moves to escape)<br>2- No response to auditory stimulus; moderate response to touch (moves a few steps)<br>3- No response to auditory stimulus; mild response to touch (no locomotion)<br>4- No response to auditory stimulus. Little or no response to touch. Cannot right itself if pushed over
Eyes	0- Open<br>1- Eyes not fully open, possibly with secretions<br>2- Eyes at least half closed, possibly with secretions<br>3- Eyes half closed or more, possibly with secretions<br>4- Eyes closed or milky
Respiration rate	0- Normal, rapid mouse respiration<br>1- Slightly decreased respiration (rate not quantifiable by eye)<br>2- Moderately reduced respiration (rate at the upper range of quantifying by eye)<br>3- Severely reduced respiration (rate easily countable by eye, 0.5 s between breaths)<br>4- Extremely reduced respiration (>1 s between breaths)
Respiration quality	0- Normal<br>1- Brief periods of laboured breathing<br>2- Laboured, no gasping<br>3- Laboured with intermittent gasps<br>4- Gasping

Supplementary data:

Original immunoblots images supplementary information of figure 6d (HSP70 /β-actin expression in lung of mice).

Bands represents HSP70 expression in the lung of mice.

The sequence of the Blots is Control; Sepsis and Sepsis+eHSP72 (repeatedly)

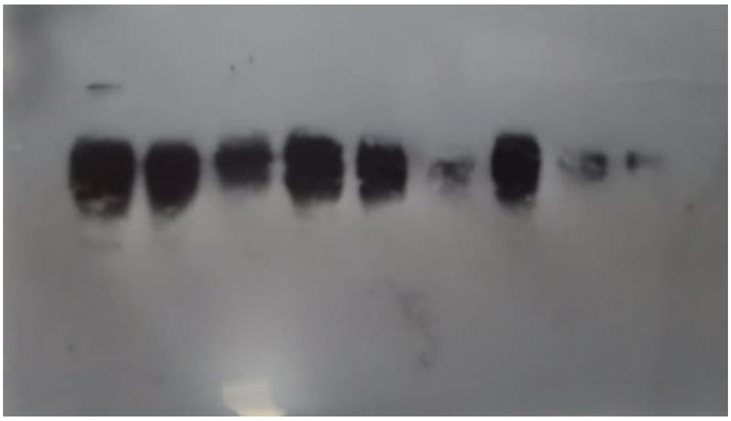

Bands represents HSP70 expression in the lung of mice.

The sequence of the Blots is Control; Sepsis and Sepsis+eHSP72 (repeatedly)

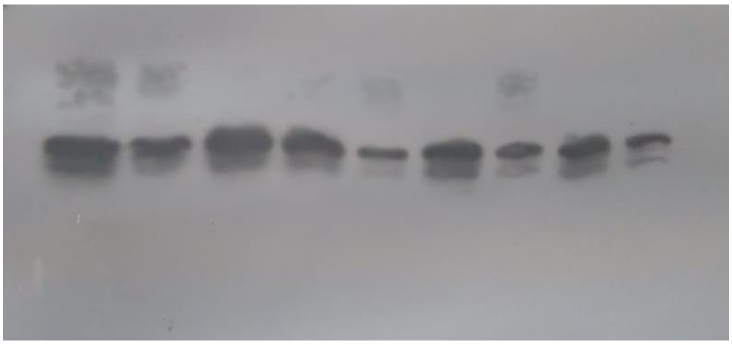

Supplement: Supplementary file 1 — Supplementary information. [file 41598_2020_66011_MOESM1_ESM.pdf]
